# Increased Oxygen Extraction by Pulmonary Rehabilitation Improves Exercise Tolerance and Ventilatory Efficiency in Advanced Chronic Obstructive Pulmonary Disease

**DOI:** 10.3390/jcm11040963

**Published:** 2022-02-12

**Authors:** Akito Miyazaki, Keisuke Miki, Ryoji Maekura, Kazuyuki Tsujino, Hisako Hashimoto, Mari Miki, Hiromi Yanagi, Taro Koba, Takuro Nii, Takanori Matsuki, Hiroshi Kida

**Affiliations:** 1 Department of Respiratory Medicine, National Hospital Organization Osaka Toneyama Medical Center, Toyonaka 560-8552, Japan; miyazaki.akito.qk@mail.hosp.go.jp (A.M.); tsujino.kazuyuki.bh@mail.hosp.go.jp (K.T.); hashimoto.hisako.cz@mail.hosp.go.jp (H.H.); koba.taro.sj@mail.hosp.go.jp (T.K.); nii.takuro.kw@mail.hosp.go.jp (T.N.); matsuki.takanori.qn@mail.hosp.go.jp (T.M.); kida.hiroshi.sv@mail.hosp.go.jp (H.K.); 2 Graduate School of Health Care Sciences, Jikei Institute, Osaka 532-0003, Japan; mae.ryoji@gmail.com; 3 Department of Internal Medicine, Tokushima Prefecture Naruto Hospital, Naruto 772-8503, Japan; mmaryys@gmail.com; 4 Department of Clinical Examination, National Hospital Organization Osaka Toneyama Medical Center, Toyonaka 560-8552, Japan; yanagi.hiromi.ex@mail.hosp.go.jp

**Keywords:** dyspnea, exercise training, oxygen uptake, ventilation

## Abstract

Background: In cardiopulmonary exercise testing (CPET), oxygen uptake (*V’*_O2_) is calculated using the product of minute ventilation (*V’*_E_) and the difference between inspiratory and expiratory O_2_ concentrations (ΔFO_2_). However, little is known about the response of ΔFO_2_ to pulmonary rehabilitation (PR). The aim of the present study was (1) to investigate whether PR increases peak *V’*_O2_, based on whether ΔFO_2_ or *V’*_E_ at peak exercise increase after PR, and (2) to investigate whether an improvement in ΔFO_2_ correlates with an improvement in ventilatory efficiency. Methods: A total of 38 patients with severe and very severe COPD, whose PR responses were evaluated by CPET, were retrospectively analyzed. Results: After PR, peak *V’*_O2_ was increased in 14 patients. The difference in ΔFO_2_ at peak exercise following PR correlated with the difference in peak *V’*_O2_ (r = 0.4884, *p* = 0.0019), the difference in *V’*_E_/*V’*_CO2_-nadir (r = −0.7057, *p* < 0.0001), and the difference in *V’*_E_–*V’*_CO2_ slope (r = −0.4578, *p* = 0.0039), but it did not correlate with the difference in peak *V’*_E_. Conclusions: The increased O_2_ extraction following PR correlated with improved exercise tolerance and ventilatory efficiency. In advanced COPD patients, a new strategy for improving O_2_ extraction ability might be effective in those in whom ventilatory ability can be only minimally increased.

## 1. Introduction

Since 2018, chronic obstructive pulmonary disease (COPD) has become the world’s third leading cause of death [1]. The most frequent and intractable problem in patients with COPD is exercise intolerance due to wasted ventilation [2,3,4,5], which leads to poor disease prognosis [6]. Although several measures have been attempted to improve exercise tolerance, they remain insufficient.

It is widely accepted that pulmonary rehabilitation (PR), serving as an effective non-pharmacological intervention for COPD, improves endurance, quality of life and exertional dyspnea [7,8]. Consequently, since the survival of patients with COPD is also improved, PR is considered a very useful treatment for COPD patients [9,10]. The response to PR, however, varies significantly between patients, and PR including exercise training does not necessarily increase peak oxygen uptake (*V’*_O2_), especially in patients with advanced COPD [11,12]. Physical exercise requires gas exchange of both oxygen (O_2_) and carbon dioxide (CO_2_) and involves the interaction of pulmonary, cardiovascular and muscle crosstalk in the body [6,13]. Hence, *V’*_O2_, as determined by cardiopulmonary exercise testing (CPET), is calculated using the product of minute ventilation (*V’*_E_), as a measure of ventilatory ability, and the difference between inspired and expired O_2_ concentrations (ΔFO_2_), as a measure of O_2_ extraction ability. Therefore, peak *V’*_O2_ is one of the variables used to characterize total exercise ability and might be informative for assessing the efficacy of PR. In addition, reduced peak *V’*_O2_ has been proven to predict a poor prognosis in patients with COPD [14,15], and effective evaluation of peak *V’*_O2_ might serve as a guide for decision-making to confirm the pathophysiological condition and choose suitable treatment for COPD.

In several chronic cardiopulmonary diseases, ventilatory inefficiency, indicated by a high *V’*_E_ versus volume of exhaled carbon dioxide (*V’*_CO2_) relationship during exercise, i.e., a high *V’*_E_–*V’*_CO2_ slope, is used as a prognostic marker and is commonly associated with a low arterial carbon dioxide partial pressure (PaCO_2_) during exercise [16,17,18]. Recently, we reported that a high *V’*_E_–*V’*_CO2_ slope was more strongly associated with a low ΔFO_2_ at peak exercise, as a gas exchange parameter related to O_2_, as compared to CO_2_-related variables, such as PaCO_2_ and partial pressure of end-tidal CO_2_ (PetCO_2_), in patients with COPD [19]. Furthermore, we reported that the dependence of reduced peak *V’*_O2_ on ΔFO_2_ becomes relatively high with the progression of COPD, due to a decrease in its dependence on ventilatory ability at peak exercise, and that increasing ΔFO_2_ might be an attractive approach for improving exercise tolerance and ventilatory efficiency, especially in advanced COPD patients [19].

The aim of the present study was: (1) to investigate whether PR increases exercise tolerance, evaluated by assessment of peak *V’*_O2_ during incremental CPET, and to assess the effect of PR based on the variables that are more significantly associated with improvement in peak *V’*_O2_, i.e., ΔFO_2_ or *V’*_E_ at peak exercise and (2) to investigate whether an improvement in ΔFO_2_ is predictive of an improvement in ventilatory efficiency, indicated by the *V’*_E_–*V’*_CO2_ slope and the lowest value of *V’*_E_/*V’*_CO2_ during exercise in patients with severe and very severe COPD.

## 2. Materials and Methods

### 2.1. Study Design

This retrospective study was conducted by analyzing data obtained from severe and very severe COPD patients who underwent PR while in hospital and were evaluated using CPET before and after PR at the National Hospital Organization (NHO) Osaka Toneyama Medical Center from April 2000 to July 2021. This study included data from previous ethically-approved studies performed as screening for studies on COPD at our institution. Thus, a total of 38 patients diagnosed and classified as stage III or IV COPD according to the Global Initiative for Chronic Obstructive Lung Disease (GOLD) criteria [20] were analyzed. Patients were excluded if they met the following criteria: (1) if they had a diagnosis of bronchial asthma, active infection, or severe heart disease, (2) had a history of lung resection, (3) if their drug regimens were changed within 4 weeks before PR, (4) if new treatment was added during PR, and (5) if their conditions were unstable due to other reasons. This study was performed in accordance with the Declaration of Helsinki and the institutional review board of the NHO Osaka Toneyama Medical Center approved this study (approval number: TNH-A-2021022). Written, informed consent was obtained from each patient before the first CPET evaluation.

### 2.2. Pulmonary Rehabilitation (PR)

Education and instruction, physical therapy, exercise training and occupational therapy were included in the PR program and performed in hospital. The patients were instructed to use educational material to increase their knowledge of the disease and to improve their management of it. They underwent three sets of exercise training per day with electromechanically braked cycle ergometers, from three to five days a week for one to two months (20 days), with high-intensity targets as previously described [21]. The initial exercise level was set for 6 min a set at the work rate equivalent to 60% of the baseline peak *V’*_O2_ before PR. If they could tolerate the exercise, the exercise duration was first increased to 10 min a set, following which the work rate was increased by 5 W and if possible, increased to the work rate equivalent to 80% of the baseline peak *V’*_O2_. If the patients could not tolerate the exercise, their exercise levels were reduced to the previous setting.

### 2.3. Pulmonary Function Tests (PFTs)

Post-bronchodilator spirometry (CHESTAC 8800; CHEST M.I. Inc., Tokyo, Japan) was performed according to the recommendations of the American Thoracic Society [22]. PFTs were performed within 1 week before and after PR.

### 2.4. Six-Minute Walk Test

The six-minute walk distance (6-MWD) was measured as described previously [23]. The patients were instructed to walk at their own pace but to cover as much ground as possible in 6 min without encouragement. 

### 2.5. Cardiopulmonary Exercise Testing (CPET)

Symptom-limited incremental exercise tests were performed using an electrically braked cycle ergometer (CV-1000SS; Lode, Groningen, The Netherlands) and a CPET system (Aero monitor AE310S; Minato Medical Science Co., Ltd., Osaka, Japan) before and after PR with the same protocol, i.e., the two-minute stage, 10-watt step protocol. Before CPET, patients were instructed to perform to their maximal effort but were advised that the exercise could be stopped at any time. During exercise, CPET was performed without encouragement until the subject was exhausted in order to achieve reliable data. All patients were instructed to maintain a speed of approximately 60 rpm on the cycle ergometer by observing the rpm meter. Resting measurements before exercise were obtained during the steady-state period after at least 3 min of rest after preparation for CPET. Ventilatory values were measured on a breath-by-breath basis using a face mask and are shown as 30-s averages at rest, at two-minute intervals during exercise and at the end of exercise. Severity of dyspnea (10-point modified Borg category-ratio scale) was evaluated at rest, during the last 15 s of each exercise stage and at the end of exercise, and all patients were asked which symptoms (exertional dyspnea, leg discomfort, or others) caused them to stop the exercise. The *V’*_E_–*V’*_CO2_ slope was calculated by linear regression, excluding the nonlinear part of the data after the onset of the respiratory compensation point. If the respiratory compensation point could not be determined, the *V’*_E_–*V’*_CO2_ slope was calculated from the data from the start to the end of the exercise. The *V’*_E_–*V*’_CO2_ nadir was defined as the lowest value of the ratio between *V’*_E_ and *V*’_CO2_ during exercise. The *V’*_E_–*V’*_CO2_ intercept was defined as the nonzero point on the *Y*-axis, i.e., *V’*_E_ [19]. The physiological dead space to tidal volume ratio (*V*_D_/*V*_T_) was estimated based on Enghoff’s modification of Bohr’s equation [17], using the non-invasive parameter of PetCO_2_ as an approximation of PaCO_2_. *V*_D_-intercept was estimated as *V’*_E_–*V’*_CO2_ intercept/*f*_R_–*V’*_CO2_ intercept during exercise, and the *f*_R_–*V’*_CO2_ intercept was defined as the nonzero point on the *Y*-axis, i.e., *f*_R_ [24]_._ The time-slope was calculated as the ratio of exercise time until exhaustion to Δ*V’*_O2_ (peak minus resting *V’*_O2_) obtained during CPET [12]. The predicted maximal voluntary ventilation (MVV) was calculated as FEV_1_ × 35. The predicted maximum heart rate was calculated as 220—age in years [13]. The percent predicted peak *V’*_O2_ was calculated using the equations of Itoh et al. [13]. Isotime was defined as the time the shortest test ended. An inflection point of *V*_T_ during exercise was determined for each subject using the 30-s averaged data [25]. The anaerobic threshold was identified using the V-slope method [13].

### 2.6. Statistical Analysis

The data are expressed as means ± standard deviation (SD). First, all patients were divided into two groups based on whether or not peak *V’*_O2_ increased after PR. Based on the results of variables with significant differences between the peak *V’*_O2_ increase group (peak *V’*_O2_ before PR< peak *V’*_O2_ after PR) and the non-increase group (peak *V’*_O2_ before PR ≥ peak *V’*_O2_ after PR), and since *V’*_O2_ is calculated using the product of *V’*_E_ and ΔFO_2_, all patients were then divided into two groups based on whether *V’*_E_ or ΔFO_2_ at peak exercise had increased after PR. That is, the increase group was defined as *V’*_E_ or ΔFO_2_ before PR was less than that after PR, and the non-increase group was defined as *V’*_E_ or ΔFO_2_ before PR was equal to or greater than that after PR. Fisher’s exact test was used to compare baseline characteristics before PR between the two groups and evaluate the reasons for stopping exercise before and after PR. Mann–Whitney’s U test was used to compare baseline characteristics before PR and compare the mean differences before and after PR between the two groups. The Wilcoxon signed-rank test was used to compare the results after PR with the results before PR within each group. Univariate analysis using Spearman’s rank correlation coefficient as a non-parametric test was used to evaluate the correlations of ΔFO_2_ at peak exercise with the other clinical variables. A *p* value < 0.05 was considered to indicate significance (JMP software, version 11, SAS Institute Inc., Cary, NC, USA).

## 3. Results

A total of 38 patients with severe to very severe airway obstruction according to the GOLD stages were evaluated before and after PR (Table 1). All patients were ex-smokers. Before PR, all patients performed incremental CPET (mean exercise time 7.0 min, maximum value 12.9 min and minimum value 2.8 min), and their mean peak *V’*_O2_ was 13.3 mL·min^−1^·kg^−1^, suggesting obvious exercise intolerance.

Although the difference following PR in six-MWD, which was evaluated as endurance, was a positive value in 81% of the patients, the difference following PR in peak *V’*_O2_ was positive in only 14 (37%) patients (Table 2). In all patients, the mean difference following PR in peak *V’*_O2_ was not increased (mean difference from pre-PR: −0.02 mL·min^−1^·kg^−1^). Of these 14 patients, ΔFO_2_ at peak exercise during CPET after PR was increased in 11 patients (79%), while in the remaining three patients, *V’*_E_ increased despite a decrease in ΔFO_2_ at peak exercise after PR. In the peak *V’*_O2_ increase group, of the CPET variables obtained, the changes in the ΔFO_2_ and *V’*_E_ at peak exercise after PR were significantly higher than in the peak *V’*_O2_ non-increase group among CPET variables obtained (Table 2).

**Figure 1:**
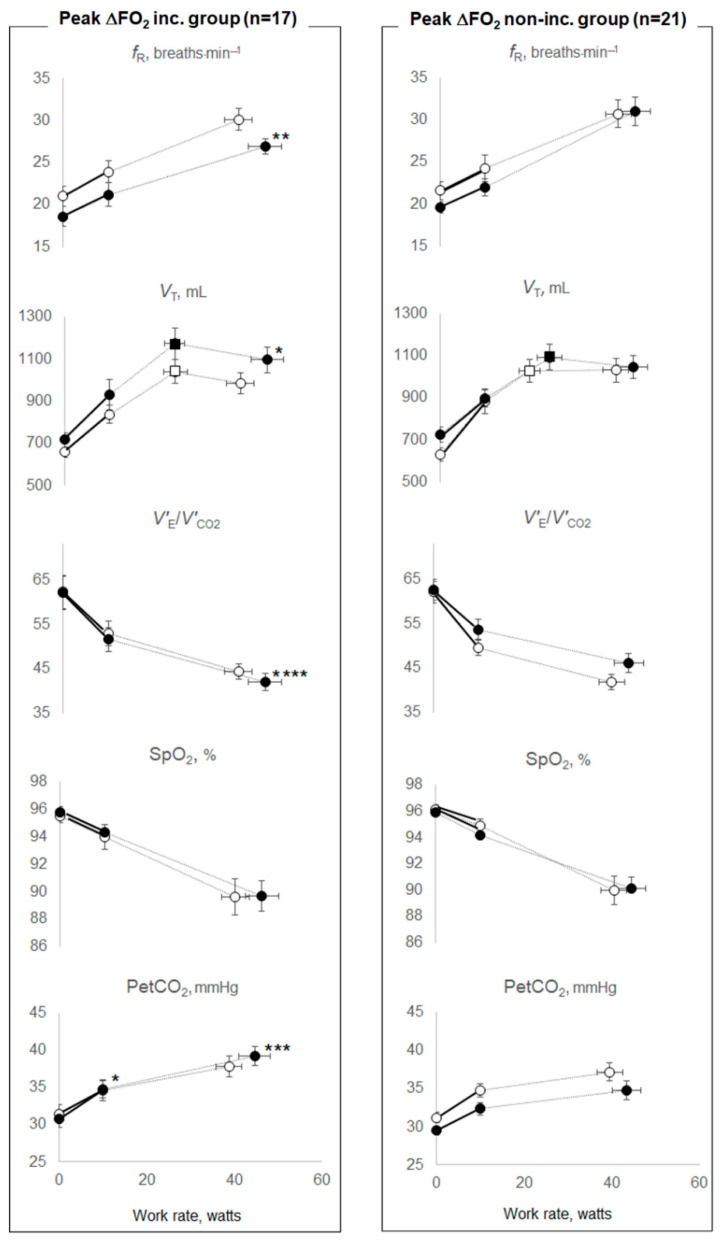
Exercise variables before and after pulmonary rehabilitation between the peak ΔFO_2_ increase and non-increase groups. Data are presented as means (standard error). All patients were divided into two groups based on whether or not ΔFO_2_ at peak exercise had increased after pulmonary rehabilitation. Peak ΔFO_2_: difference between inspiratory and expiratory O_2_ concentrations at peak exercise; inc.: increase; *f*_R_: breathing frequency; *V*_T_: tidal volume; *V’*_E_: minute ventilation; *V’*_CO2_: carbon dioxide output; SpO_2_: percutaneous oxygen saturation; PetCO_2_: partial pressure of end-tidal carbon dioxide. * *p* < 0.05, ** *p* < 0.01, *** *p* < 0.001, **** *p* < 0.0001: mean differences between pre-and post-pulmonary rehabilitation values at each exercise phase were compared between peak ΔFO_2_ increase and non-increase groups. Open symbols: before pulmonary rehabilitation. Closed symbols: after pulmonary rehabilitation. Squares represent *V*_T_–ventilation inflection points.

**Figure 2:**
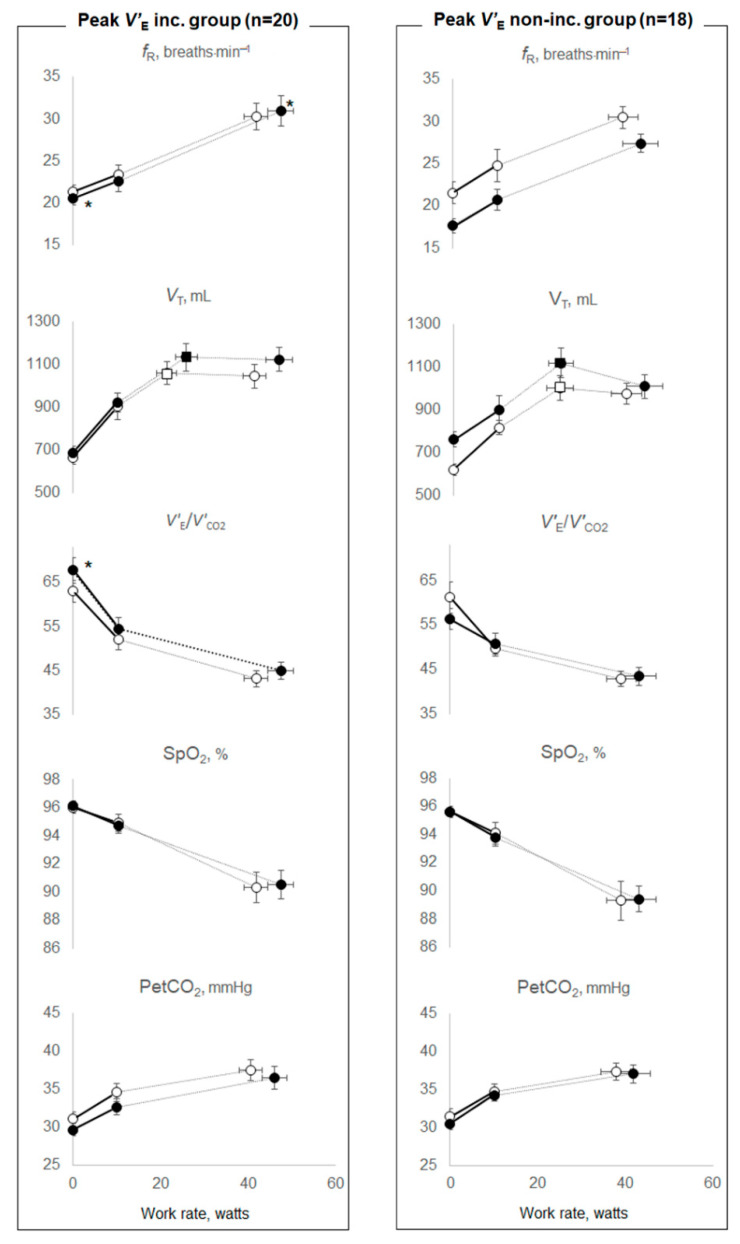
Exercise variables before and after pulmonary rehabilitation between the peak *V’*_E_ increase and non-increase groups. Data are presented as means (standard error). All patients were divided into two groups based on whether or not *V’*_E_ at peak exercise had increased after pulmonary rehabilitation. *V’*_E_: minute ventilation; inc.: increase; *f*_R_: breathing frequency; *V*_T_: tidal volume; *V’*_CO2_: carbon dioxide output; SpO_2_: percutaneous oxygen saturation; PetCO_2_: partial pressure of end-tidal carbon dioxide. * *p* < 0.05: mean differences between pre-and post-pulmonary rehabilitation values at each exercise phase were compared between peak *V’*_E_ increase and non-increase groups. Open symbols: before pulmonary rehabilitation. Closed symbols: after pulmonary rehabilitation. Squares represent *V*_T_–ventilation inflection points.

Therefore, we investigated the effects of PR on O_2_ extraction or ventilatory ability by dividing the patient cohort into two groups based on whether or not they experienced an increase in the ΔFO_2_ or *V’*_E_ at peak exercise after PR (Table 3, Table 4 and Table 5). The groups divided according to the increase in the ΔFO_2_ or *V’*_E_ at peak exercise were well matched for age, sex and body mass index (BMI), GOLD stages and resting pulmonary function, although the number of subjects with dual or triple inhalation therapy was low in each group (Table 3). In the peak ΔFO_2_ increase group, compared with the change following PR in the peak ΔFO_2_ non-increase group, (1) the mean differences following PR in peak *V’*_O2_ (*p* = 0.0136), *V’*_O2_ at anaerobic threshold (*p* < 0.0001), and PetCO_2_ at peak exercise (*p* = 0.0007) were significantly increased, and those in *V’*_E_/*V’*_CO2_-nadir (*p* < 0.0001) during exercise and *V’*_E_–*V’*_CO2_-slope (*p* = 0.0413) were significantly improved, although *V’*_E_–*V’*_CO2_ intercept was not changed (Table 4), (2) notwithstanding that the mean difference following PR in *V*_T_ at peak exercise was increased (*p* = 0.0167), and in the *f*_R_ at peak exercise was reduced (*p* = 0.0049), the mean difference following PR in the inspiratory duty cycle (Ti/Ttot) at peak exercise (*p* = 0.4788) and in *V*_T_ at the inflection point during exercise (*p* = 0.1798) were not changed (Table 4 and Figure 1). Then, the mean difference following PR in *V’*_E_ did not increase significantly (*p* = 0.7134) (Table 4), and (3) the mean difference following PR in O_2_-pulse was not significantly changed (*p* = 0.2218) (Table 4). In the peak ΔFO_2_ non-increase group, (i) dyspnea at peak exercise was significantly reduced, as seen in the within-group evaluation (*p* = 0.0006), (ii) the time-slope was significantly lower before PR (*p* = 0.0277) and increased after PR compared with the peak ΔFO_2_ increase group (*p* = 0.0302) (Table 4). In contrast, in the peak *V’*_E_ increase group, compared with the mean difference following PR in the peak *V’*_E_ non-increase group, although the mean difference following PR in peak *V’*_O2_ was significantly increased (*p* = 0.0109), those in *V’*_O2_ at the anaerobic threshold (*p* = 0.6429), in *V’*_E_/*V’*_CO2_-nadir during exercise (*p* = 0.5685), in *V’*_E_–*V’*_CO2_-slope (*p* = 1.0000), in ΔFO_2_ (*p* = 0.4558)_,_ and in PetCO_2_ (*p* = 0.5200) at peak exercise did not change, and significant tachypnea at peak exercise (*p* = 0.0415) was seen (Table 5 and Figure 2).

**Figure 3:**
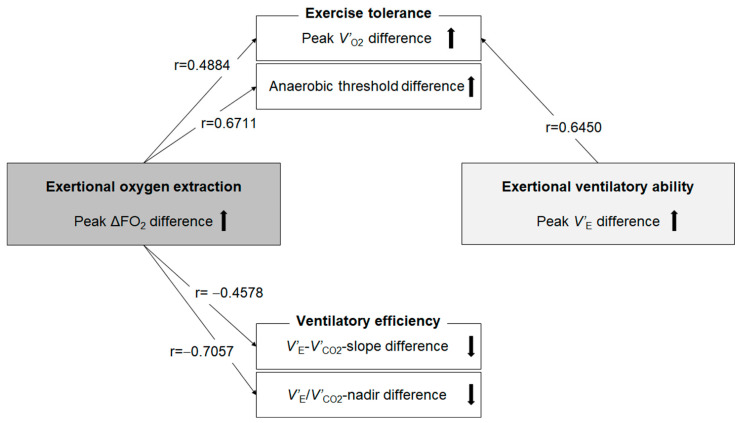
Correlations related to exercise tolerance and ventilatory efficiency. Difference: between before and after pulmonary rehabilitation; ΔFO_2_: difference between inspired and expired O_2_ concentration; *V’*_E_: minute ventilation; *V’*_E_/*V’*_CO2_-nadir: lowest value of the ratio between minute ventilation and carbon dioxide output during exercise (see the Methods section for details); *V’*_E_–*V’*_CO2_-slope: the slope was determined by linear regression analysis of minute ventilation to carbon dioxide output observed during exercise (see the Methods section for details); *V’*_O2:_ oxygen uptake. Black arrows mean the up and down differences obtained from pulmonary rehabilitation.

Next, we investigated whether the change in ΔFO_2_ at peak exercise following PR correlated with changes in the other CPET variables, as shown in Table 6 and Figure 3. The difference in ΔFO_2_ at peak exercise resulting from PR correlated positively with the difference following PR in peak *V’*_O2_ (r = 0.4884, *p* = 0.0019) and *V’*_O2_ at the anaerobic threshold (r = 0.6711, *p* = 0.0001) and correlated negatively with the difference following PR in *f*_R_ (r = −0.3894, *p* = 0.0157), the difference in *V’*_E_/*V’*_CO2_-nadir during exercise (r = −0.7057, *p* < 0.0001) and the difference in *V’*_E_–*V’*_CO2_-slope (r = −0.4578, *p* = 0.0039). The change in PetCO_2_ at peak exercise following PR correlated with the difference in *V’*_E_/*V’*_CO2_-nadir during exercise (r = −0.5227, *p* < 0.0001) and the difference in *V’*_E_–*V’*_CO2_-slope (r = −0.3448, *p* = 0.0340), although the significance of this was lower than the correlation with the change in ΔFO_2_. In addition, the change in ΔFO_2_ at peak exercise following PR was positively correlated with the time-slope before PR (r = 0.4120, *p* = 0.0102). No significant correlation was confirmed between the change in *V’*_E_ at peak exercise and the change in *V’*_E_/*V’*_CO2_-nadir during exercise (r = 0.0795, *p* = 0.6352), and the change in *V’*_E_–*V’*_CO2_-slope (r = 0.0915, *p* = 0.5850) or between the change in *V’*_E_ and the change in ΔFO_2_ at peak exercise following PR (r = −0.0988, *p* = 0.5552) (Table 6).

## 4. Discussion

The present study aimed to investigate whether PR leads to an increase in incremental effort evaluated by CPET and whether the resultant change in O_2_ extraction affects the exertional pathophysiological conditions after PR in advanced COPD patients. The main observations were as follows. First, peak *V’*_O2_ was increased in only 14 (37%) patients. Second, in the peak ΔFO_2_ increase group (17 of 38 patients), exercise tolerance and ventilatory efficiency were improved by PR without an increase of *V’*_E_. In the peak *V’*_E_ increase group (20 of 38 patients), although peak *V’*_O2_ was significantly increased, ventilatory efficiency did not improve. Third, in all the patients, the difference in O_2_ extraction at peak exercise before and after PR correlated positively with the difference in exercise tolerance, and negatively with the difference in ventilatory efficiency.

Increasing O_2_ extraction, which is evaluated as ΔFO_2_ in CPET, would help improve exercise tolerance, including the anaerobic threshold and ventilatory efficiency, particularly in patients with advanced COPD (Figure 3). Based on the Fick principle, *V’*_O2_ is the product of cardiac output and the difference between arterial and mixed venous oxygen content. The difference in arteriovenous oxygen content reflects O_2_ extraction by the muscles [26]. In CPET, only ventilatory flow and the inspired and expired concentrations of O_2_ and CO_2_ are directly measured at the mouth; all other variables are calculated using these measurements. In CPET, *V’*_O2_ is calculated using the product of *V’*_E_ and ΔFO_2_ [6,13]. The latter reflects total O_2_ extraction related to cardiopulmonary–muscle crosstalk in the body. Of note, ΔFO_2_ did not correlate with *V’*_E_ at peak exercise in our previous study [19]. Furthermore, the change in ΔFO_2_ at peak exercise following PR did not correlate with the change in *V’*_E_ after PR in the present study (Table 6). These findings suggest that ΔFO_2_ might be usable for CPET evaluations independent of *V’*_E_. However, little is known about whether this total O_2_ extraction affects exertional pathophysiological conditions including airflow limitation, cardiac dysfunction and metabolic changes in COPD patients. In the peak ΔFO_2_ increase and non-increase groups, no significant treatment changes in *V*_T_ at the inflection point during exercise, in Ti/Ttot at peak exercise and in O_2_-pulse, i.e., the product of the stroke volume and arterial–venous O_2_ content difference, were observed (Table 4 and Figure 1); that is, the changed levels following PR of the mechanical constraints on *V*_T_ and the prolonged expiration pattern during exercise due to wasted ventilation and the changed levels of cardiac dysfunction were similar between the two groups. In addition, the difference in ΔFO_2_ at peak exercise resulting from PR correlated with the difference following PR in peak *V’*_O2_ and the anaerobic threshold (Table 6 and Figure 3), but it did not correlate with the difference following PR in *V’*_E_ or O_2_-pulse at peak exercise (Table 6). The response that the higher oxygen extraction obtained from PR improved exercise tolerance shifting the anaerobic threshold point to the late exercise phase might be caused by improved muscle condition related to O_2_ extraction rather than a direct cardiopulmonary mechanism in the cardiopulmonary-muscle crosstalk. Our previous report [19] and the present study demonstrated that ΔFO_2_ at peak exercise and the change in ΔFO_2_ following PR, which is a gas exchange parameter related to O_2_, rather than CO_2_-related variables, such as PetCO_2_, had a stronger inverse relationship with *V’*_E_–*V’*_CO2_ slope and the change in the *V’*_E_–*V’*_CO2_ slope following PR, respectively. These findings suggest that the *V’*_E_–*V’*_CO2_ slope is a comprehensive variable that reflects not only CO_2_ gas exchangeability, but also O_2_ extraction ability. Not only that, they might illustrate the response, as shown in Figure 3, that higher oxygen extraction from PR had on improving ventilatory efficiency, as well as shifting the highest *V’*_O2_ point without developing an exertional acidosis to the late exercise phase, and they were associating with each other. The response might be the reason why *f*_R_ at peak exercise was reduced after PR in the peak ΔFO_2_ increase group (Table 4). In contrast, in the peak *V’*_E_ increase group, peak *V’*_O2_ increased, but the increase in *V’*_E_ after PR depended on tachypnea, and ventilatory efficiency was not improved after PR (Table 5 and Figure 2). Given the evidence that *V’*_E_–*V’*_CO2_ slope is a prognostic factor in several chronic cardiopulmonary diseases independent of other exercise-related variables such as peak *V’*_O2_ [18], the present results suggest that much attention should be paid to the clinical information about O_2_ extraction. In terms of pre-PR parameters, only the time-slope in the peak ΔFO_2_ increase group was higher than that in the peak ΔFO_2_ non-increase group. In addition, the change in ΔFO_2_ at peak exercise resulting from PR was positively correlated with the time-slope before PR. These findings indicate that the more gently patients exercised to obtain a certain *V’*_O2_ before PR_,_ the larger was the change in O_2_ extraction obtained after PR. Furthermore, in our previous study, the ΔFO_2_–*V’*_CO2_ slope during exercise correlated positively with the PaCO_2_–*V’*_CO2_ slope, that is, the degree of exertional respiratory acidosis in COPD patients [19]. Although a slower rate of change in *V’*_O2_ for a given change in work rate is generally recognized in CPET responses, indicating poor O_2_ delivery or extraction [13], these findings suggest that increasing O_2_ extraction would not only improve exercise tolerance and ventilatory efficiency but might also play a compensatory or protective role during exercise in advanced COPD patients.

Improving exercise tolerance, especially incremental effort, following PR might be difficult in some patients, particularly in those with advanced COPD [11,12], although various strategies for improving exercise tolerance in COPD patients have been studied. Surprisingly, in the present study, PR resulted in a decrease in dyspnea at peak exercise even in the peak ΔFO_2_ non-increase group, despite the lack of improvement in incremental effort. This could be explained by the assumption that less incremental effort required for exercise resulting from PR might have led to a decrease in dyspnea reducing O_2_ extraction because exercise tolerance and ventilatory efficiency could not improve sufficiently following PR. Meanwhile, given that international position statements recommend that PR programs should offer exercise training for 8–10 weeks, which is longer than the duration of PR in the present study [7], we speculate that a longer PR program might increase ΔFO_2_, leading to improved exercise tolerance and hence might be needed in certain advanced COPD patients in whom an inability to improve incremental effort is expected. Though the investigation did not specifically address for treatment change in ΔFO_2_ evaluated by CPET, we fortunately found that oxygen extraction was improved by the administration of ghrelin [27], which was first discovered to have a variety of effects including direct effects of vasodilation [28] and an increase in cardiac output [29]. We previously reported that activated ghrelin (acyl ghrelin) treatment without PR improved peak *V’*_O2_ in patients with severe and very severe COPD and that this effect might be attributed to the resultant improvements in cardiac function by O_2_ pulse and an increase in ΔFO_2_ rather than *V’*_E_ [30]. Thus, developing appropriate strategies for improving ΔFO_2_, using the interaction of the pulmonary, cardiovascular and muscle crosstalk in the body, might lead to a clear understanding of the mechanism by which increasing O_2_ extraction improves exercise tolerance and gas exchange in COPD patients.

The present study has some limitations. First, this was a single-center study with a small number of patients, and the number of female patients was disproportionately low. Second, detailed evaluation of O_2_ delivery–utilization in skeletal muscles was not performed in the present study, although it has been reported that the skeletal muscle area measured by computed tomography correlates with ventilatory efficiency during CPET [31], and hence, would have correlated with O_2_ extraction during exercise in the present study and our previous study [19]. Third, the study included some COPD patients who regularly took short-acting muscarinic antagonists rather than long-acting muscarinic antagonists, and the number of patients receiving dual or triple therapy including inhalation therapy was low, which might have affected the results. Fourth, blood samples for blood gas analyses were not collected during CPET for the estimation of dead space values during exercise and elucidation of the pathophysiological mechanisms during exercise. Admittedly, two non-invasive estimations of dead space volume were used, but in both the peak ΔFO_2_ or *V’*_E_ increase and non-increase groups, no significant differences in the mean change following PR in *V*_D_/*V*_T_ estimated by Enghoff’s modification of Bohr’s equation and *V*_D_-intercept/*V*_T_ were observed (Table 4 and Table 5). Fifth, given that the optimal test duration for CPET should be from eight to twelve minutes [13,32], the relatively short test duration in the present study might have affected the results, especially for the calculations using exertional ventilatory variables vs. *V’*_CO2_ relationship, such as the slope and *Y*-axis intercept. Sixth, no significant difference in improvement in exertional dyspnea at peak exercise was observed between the peak ΔFO_2_ increase and non-increase groups. In addition, isotime comparisons showed that Borg scale scores tended to decrease after PR (mean Borg scale score, pre-PR 6.1 ± 2.9, post-PR 4.4 ± 2.9; within-group comparison, *p* = 0.0797) in the peak ΔFO_2_ increase group, indicating that the exertional dyspnea was reduced at isotime. However, the change was not significantly different between the peak ΔFO_2_ increase and non-increase groups (*p* = 0.6177). The resultant endurance effort rather than the incremental effort obtained from PR might affect the decrease in exertional dyspnea in the peak ΔFO_2_ non-increase group, although it may be difficult to distinguish the effect of endurance effort if an incremental effort was not obtained from PR. Further studies including a specific treatment strategy targeting oxygen extraction might be necessary to confirm whether increasing oxygen extraction is useful for improving exertional dyspnea.

## 5. Conclusions

PR resulted in increased exercise tolerance in only 37% of patients with advanced COPD in the present study. The change in O_2_ extraction ability resulting from PR, evaluated as the ΔFO_2_ during exercise, correlated positively with the post-PR change in exercise tolerance and the anaerobic threshold, and negatively with the change in ventilatory efficiency. In patients with advanced COPD, it is often difficult to increase ventilatory ability because of the dynamically hyperinflated lungs. Hence, new strategies involving the improvement of O_2_ extraction ability are needed for better exercise tolerance and improved ventilatory efficiency.

## Figures and Tables

**Table 1:** Patient baseline characteristics (*n* = 38).

	All Patients (*n* = 38)
Age, years	68.9 (9.1)
Sex, male/female	37/1
BMI, kg·m^−2^	20.1 (3.7)
GOLD stage, III/IV	21/17
Pulmonary function	
FEV_1_, L	0.84 (0.29)
%FEV_1_, %predicted	31.6 (10.3)
FEV_1_/FVC, %	39.5 (9.2)
VC, L	2.69 (0.62)
%VC, %predicted	83.3 (16.9)
IC, L	1.71 (0.46)
6-MWD *, m	262.8 (113.3)
Incremental CPET	
Peak *V’*_O2_, mL·min^−1^·kg^−1^	13.3 (3.7)
Percent predicted peak *V’*_O2_, %	57.8 (16.4)
SAMA	17
LAMA	9
SABA	6
LABA	14
ICS	9
LAMA·LABA/ICS·LABA/Triple therapy	7/4/1

Data are presented as means (standard deviation) unless otherwise specified. BMI: body mass index; GOLD: Global Initiative for Chronic Obstructive Lung Disease; FEV_1_: forced expiratory volume in one second; FVC: forced vital capacity; VC: vital capacity; IC: inspiratory capacity; six-MWD: six-minute walk distance; CPET: cardiopulmonary exercise testing; *V’*_O2_: oxygen uptake; SAMA: short-acting muscarinic antagonist; LAMA: long-acting muscarinic antagonist; SABA: short-acting β_2_-agonist; LABA: long-acting β_2_-agonist; ICS: inhaled corticosteroid. * The data of 6-MWD were not obtained from two patients.

**Table 2:** Changes in cardiopulmonary variables after pulmonary rehabilitation in the peak *V’*_O2_ increase and peak *V’*_O2_ non-increase groups (*n* = 38).

	Peak *V’*_O2_ Inc. Group (*n* = 14)		Peak *V’*_O2_ Non-Inc. Group (*n* = 24)		*p*-Value (Between the Two Groups)
	Pre-PR	Difference	Pre-PR	Difference	
Pulmonary function					
FEV_1_, L	0.77 (0.26)	+0.16 (0.19) *	0.87 (0.30)	+0.02 (0.13)	0.0514
IC, L	1.58 (0.42)	+0.16 (0.34)	1.78 (0.47)	−0.10 (0.33)	0.0735
6-MWD ^†^, m	292.3 (104.6)	+43.2 (53.6) **	243.2 (117.0)	+56.8 (65.8) ***	0.6373
Incremental CPET at peak exercise					
Dyspnea, Borg scale	6.6 (2.5)	−1.1 (2.0)	6.3 (2.6)	−1.3 (2.2) **	0.8299
Work rate, watts	39 (9)	+9 (8) **	39 (14)	+2 (10)	0.0183
*V’*_O2_ at anaerobic threshold ^††^, mL·min^−1^	521.5 (152.6)	+33.6 (52.6)	547.4 (80.0)	−31.4(51.5) *	0.0045
R	1.04 (0.08)	+0.03 (0.08)	1.03 (0.11)	−0.01 (0.05)	0.0863
*V’*_E_, L·min^−1^	29.3 (7.4)	+4.0 (7.3) **	29.8 (6.6)	−1.2 (2.5) *	0.0008
*V*_T_, mL	986 (182)	+133 (154) ***	1025 (258)	+15 (153)	0.0062
*f*_R_, breaths·min^−1^	31 (8)	−1 (6)	30 (5)	−1 (6)	0.9516
T_i_/T_tot_	0.37 (0.04)	+0.01 (0.04)	0. 36 (0.07)	+0.02 (0.05)	0.7038
*V*_D_/*V*_T_	0.37 (0.08)	−0.02 (0.04)	0.35 (0.06)	+0.02 (0.05) *	0.0185
HR, beats·min^−1^	116 (18)	+6 (10)	119 (20)	+ 1 (16)	0.1112
O_2_ pulse, mL·beats^−1^	6.7 (1.2)	+0.4 (0.7) *	6.0 (1.5)	−0.5 (0.5) ****	0.0001
SpO_2_, %	90.4 (4.6)	0 (2.5)	89.5 (5.5)	−0.6 (3.2)	0.4565
PetCO_2_, mmHg	37.8 (6.7)	+0.4 (3.2)	37.3 (4.9)	−1.3 (3.5)	0.1463
ΔFO_2,_ %	2.79 (0.50)	+0.15 (0.33)	2.91 (0.42)	−0.20 (0.30) **	0.0037
*V*_D_-intercept/*V*_T_	0.46 (0.20)	−0.01 (0.41)	0.40 (0.25)	+0.01 (0.23)	0.5701
*V’*_E_/*V’*_CO2_	43.8 (7.8)	-1.1 (4.2)	42.5 (7.6)	+2.6 (4.2) **	0.0168
*V’*_E_/*V’*_CO2_-nadir	43.3 (8.0)	−1.4 (3.1)	42.3 (7.6)	+2.4 (4.2) **	0.0062
*V’*_E_−*V’*_CO2_-slope	31.7 (7.2)	+1.3 (4.0)	34.3 (10.2)	+3.3 (4.7) **	0.3884

Data are presented as means (standard deviation). *V’*_O2_: oxygen uptake; inc.: increase; PR: pulmonary rehabilitation; FEV_1_: forced expiratory volume in one second; IC: inspiratory capacity; 6-MWD: six-minute walk distance; CPET: cardiopulmonary exercise testing; R: gas exchange ratio; *V’*_E_: minute ventilation; *V*_T_: tidal volume; *f*_R_: breathing frequency; T_i_/T_tot_: inspiratory duty cycle; *V*_D_/*V*_T_: physiological dead space/tidal volume ratio; HR: heart rate; SpO_2_: percutaneous oxygen saturation; PetCO_2_: partial pressure of end-tidal carbon dioxide; ΔFO_2_: difference between inspiratory and expiratory O_2_ concentrations; *V*_D_-intercept: *V*_D_ calculated as *V’*_E_-axis intercept/*f*_R_-axis intercept, obtained from *V’*_E_ vs. *V’*_CO2_ and *f*_R_ vs. *V’*_CO2_ relationships (see the Methods section for details); *V’*_CO2_: carbon dioxide output; *V’*_E_/*V’*_CO2_-nadir: lowest value of the ratio between *V’*_E_ and *V’*_CO2_ during exercise (see the Methods section for details); *V’*_E_–*V’*_CO2_-slope: the slope was determined by linear regression analysis of *V’*_E_ to *V’*_CO2_ relationship (see the Methods for details). * *p* < 0.05, ** *p* < 0.01, *** *p* < 0.001, **** *p* < 0.0001: compared with pre-PR values (within-group difference). ^†^ The data of six-MWD from 2 patients were not obtained. ^††^: 27 patients (peak *V’*_O2_ inc. group, *n* = 11; peak *V’*_O2_ non-increase group, *n* = 16), whose anaerobic thresholds were obtained before and after pulmonary rehabilitation were analyzed.

**Table 3:** Comparison between groups stratified according to the change in peak ΔFO_2_ or peak *V’*_E_ (*n* = 38) after pulmonary rehabilitation.

	Peak ΔFO_2_ Inc. Group (*n* = 17)	Peak ΔFO_2_ Non-Inc. Group (*n* = 21)	*p*-Value
Age, years	71.4 (8.5)	66.8 (9.2)	0.1301
Sex, male/female	16/1	21/0	0.2600
BMI, kg·m^−2^	19.7 (3.1)	20.5 (4.2)	0.6918
GOLD stage, III/IV	9/8	12/9	0.7956
Pulmonary function			
FEV_1_, L	0.78 (0.27)	0.88 (0.30)	0.3250
%FEV_1_, %predicted	30.6 (10.2)	32.4 (10.5)	0.6281
FEV_1_/FVC, %	41.0 (7.0)	38.3 (10.6)	0.3041
VC, L	2.57 (0.62)	2.77 (0.62)	0.2838
%VC, %predicted	81.8 (17.3)	84.5 (16.8)	0.6073
IC, L	1.57 (0.41)	1.81 (0.48)	0.1487
Incremental CPET			
peak *V’*_O2_, mL·min^−1^·kg^−1^	13.0 (4.1)	13.6 (3.3)	0.6073
LAMA·LABA/ICS·LABA/Triple	2/3/0	5/1/1	0.3836
	**Peak *V’*_E_ Inc. group (*n* = 20)**	**Peak *V’*_E_ Non-Inc. group (*n* = 18)**	***p*-Value**
Age, years	67.5 (10.0)	70.4 (8.0)	0.3960
Sex, male/female	19/1	18/0	0.3363
BMI, kg·m^−2^	20.6 (4.7)	19.7 (2.4)	0.6295
GOLD stage, III/IV	12/8	9/9	0.5359
Pulmonary function			
FEV_1_, L	0.88 (0.30)	0.78 (0.27)	0.2856
%FEV_1_, %predicted	33.1 (10.2)	30.0 (10.4)	0.4047
FEV_1_/FVC, %	40.6 (9.4)	38.2 (9.0)	0.5296
VC, L	2.79 (0.66)	2.55 (0.56)	0.2193
%VC, %	86.5 (17.5)	79.7 (15.8)	0.1561
IC, L	1.74 (0.51)	1.68 (0.42)	0.6808
Incremental CPET			
peak *V’*_O2_, mL·min^−1^·kg^−1^	13.1 (3.6)	13.6 (3.9)	0.7366
LAMA·LABA/ICS·LABA/Triple	1/3/1	6/1/0	0.0855

Data are presented as means (standard deviation) unless otherwise specified. ΔFO_2_: difference between inspiratory and expiratory O_2_ concentrations; *V’*_E_: minute ventilation; inc.: increase; BMI: body mass index; GOLD: Global Initiative for Chronic Obstructive Lung Disease; FEV_1_: forced expiratory volume in one second; FVC: forced vital capacity; VC: vital capacity; IC: inspiratory capacity; CPET: cardiopulmonary exercise testing; *V’*_O2_: oxygen uptake; LAMA: long-acting muscarinic antagonist; LABA: long-acting β_2_-agonist; ICS: inhaled corticosteroid.

**Table 4:** Changes in the results of exercise testing after pulmonary rehabilitation in the peak ΔFO_2_ increase and non-increase groups.

	Peak ΔFO_2_ Inc. Group (*n* = 17)		Peak ΔFO_2_ Non-Inc. Group (*n* = 21)		*p*-Value (Between the Two Groups)
	Pre-PR	Difference	Pre-PR	Difference	
6-MWD ^‡^, m	265.1 (128.4)	+52.2 (70.7)	260.7 (100.7)	+50.6 (51.8)	0.4779
Incremental CPET at peak exercise					
Dyspnea, Borg scale	6.3 (2.9)	−0.8 (2.4)	6.5 (2.3)	−1.6 (1.8) ***	0.2457
*V’*_O__2_, ml·min^−1^·kg^−1^	13.0 (4.1)	+0.7 (1.7)	13.6 (3.3)	−0.6 (2.2)	0.0136
Exercise time, sec	416 (154)	+58 (72) **	418 (160)	+51 (113)	0.8717
Work rate, watts	39 (12)	+6 (7) **	40 (13)	+4 (12)	0.5296
*V’*_O2_ at anaerobic threshold ^††^, mL·min^−1^	502.3 (111.8)	+50.3 (38.7) ***	564.5 (110.3)	−49.1 (30.0) ****	<0.0001
R	1.02 (0.10)	+0.02 (0.06)	1.03 (0.10)	+0.00 (0.08)	0.3104
*V’*_E_, L·min^−1^	29.2 (8.0)	−0.1 (2.7)	29.9 (5.9)	+1.4 (6.8)	0.7134
*V*_T_, mL	986 (206)	+110 (153) **	1031 (253)	+16 (159)	0.0167
*V*_T_ at inflection point, mL	1040 (204)	+155 (195) *	1029 (231)	+76 (194)	0.1798
*f*_R_, breaths·min^−1^	30 (5)	−3 (4) **	31 (7)	0 (7)	0.0049
T_i_/T_tot_	0.37 (0.05)	0 (0.04)	0.36 (0.07)	+0.02 (0.05)	0.4788
*V*_D_/*V*_T_	0.38 (0.06)	−0.01(0.04)	0.34 (0.07)	+0.02 (0.05) *	0.0534
HR, beats·min^−1^	113 (20)	+6 (9) *	122 (18)	0 (17)	0.2210
O_2_ pulse, mL·beats^−1^	5.8 (1.5)	−0.1 (0.6)	5.9 (1.4)	−0.3 (0.8) *	0.2218
SpO_2_, %	89.6 (5.4)	−0.9 (2.5)	90.0 (5.0)	+0.0 (3.3)	0.3656
PetCO_2_, mmHg	37.8 (5.8)	+1.4 (3.0)	37.2 (5.5)	−2.4 (2.8) ***	0.0007
*V’*_E_/*V’*_CO2_	44.4 (7.1)	−2.4 (2.8) **	418 (8.0)	+4.2 (3.4) ****	<0.0001
*V’*_E_–*V’*_CO2_-intercept, L·min^−1^	6.6 (2.6)	−0.5 (1.6)	6.0 (3.4)	−0.8 (2.7)	0.9298
*V*_D_-intercept/*V*_T_	0.44 (0.26)	+0.10 (0.36)	0.41 (0.20)	−0.07 (0.24)	0.4738
*V’*_E_/*V’*_CO2_-nadir	44.0 (7.4)	−2.1 (2.6) **	41.6 (7.9)	+3.5 (3.5) ****	<0.0001
*V’*_E_–*V’*_CO2_-slope	32.5 (6.7)	+0.8 (3.9)	34.0 (10.9)	+4.1 (4.5) ***	0.0413
Time slope, sec·mL^−1^·min	1.04 (0.29) ^†^	+0.02 (0.33)	0.91 (0.30)	+0.20 (0.20) ***	0.0302
The causes to stop during CPET	Pre-PR	Post-PR	Pre-PR	Post-PR	Not evaluated
Dyspnea/leg fatigue	10/7	9/8	15/6	11/10 *	Not evaluated

Data are presented as means (standard deviation). Peak ΔFO_2_: difference between inspiratory and expiratory O_2_ concentrations at peak exercise; inc.: increase; PR: pulmonary rehabilitation; six-MWD: six-minute walk distance; CPET: cardiopulmonary exercise testing; *V’*_O2_: oxygen uptake; R: gas exchange ratio; *V’*_E_: minute ventilation; *V*_T_: tidal volume; *f*_R_: breathing frequency; T_i_/T_tot_: inspiratory duty cycle; *V*_D_/*V*_T_: physiological dead space/tidal volume ratio; HR: heart rate; O_2_ pulse: *V’*_O2_/HR; SpO_2_: percutaneous oxygen saturation; PetCO_2_: partial pressure of end-tidal carbon dioxide; *V’*_CO2_: carbon dioxide output; *V*_D_-intercept: *V*_D_ calculated as *V’*_E_-axis intercept/*f*_R_-axis intercept, obtained from *V’*_E_ vs. *V’*_CO2_ and *f*_R_ vs. *V’*_CO2_ relationships (see the Methods section for details); *V’*_E_/*V’*_CO2_-nadir: lowest value of the ratio between *V’*_E_ and *V’*_CO2_ during exercise (see the Methods section for details); *V’*_E_–*V’*_CO2_-slope: the slope was determined by linear regression analysis of *V’*_E_ to *V’*_CO2_ obtained during exercise (see the Methods section for details); Time slope: ratio of exercise time until exhaustion to Δ*V’*_O2_ (peak minus resting *V’*_O2_) during CPET (see the Methods section for details). * *p* < 0.05, ** *p* < 0.01, *** *p* < 0.001, **** *p* < 0.0001: compared with pre-PR values (within-group difference). ^†^ *p* < 0.05: compared with pre-PR values between the two groups. ^‡^ The data of 6-MWD were not obtained from two patients. ^††^: 27 patients (peak ΔFO_2_ inc. group, *n* = 12; peak ΔFO_2_ non-increase group, *n* = 15), whose anaerobic thresholds were obtained before and after pulmonary rehabilitation were analyzed.

**Table 5:** Changes in the results of exercise testing after pulmonary rehabilitation in the peak *V’*_E_ increase and non-increase groups.

	Peak *V’*_E_ Inc. Group (*n* = 20)		Peak *V’*_E_ Non-Inc. Group (*n* = 18)		*p*-Value (Between the Two Groups)
	Pre-PR	Difference	Pre-PR	Difference	
6-MWD ^†^, m	268.9 (102.8)	+43.7 (55.1)	256.4 (126.4)	+59.5 (67.0)	0.5860
Incremental CPET at peak exercise					
Dyspnea, Borg scale	7.3 (2.0)	−1.8 (2.1) ***	5.5 (2.8)	−0.6 (2.0)	0.0778
*V’*_O__2_, ml·min^−1^·kg^−1^	13.1 (3.6)	+0.8 (2.3)	13.6 (3.9)	−0.9 (1.4) **	0.0109
Exercise time, sec	422 (147)	+77 (110) **	411 (167)	+29 (72) *	0.1077
Work rate, watts	41 (11)	+6 (11)	38 (14)	+4 (8)	0.8725
*V’*_O2_ at anaerobic threshold ^††^, mL·min^−1^	536.0 (124.6)	−1.7 (64.1)	538.0 (102.7)	−9.0 (58.1)	0.6429
R	1.04 (0.10)	+0.02 (0.07)	1.02 (0.11)	−0.01 (0.06)	0.3404
*V*_T_, mL	1043 (251)	+79 (160) *	976 (209)	+35 (165)	0.1932
*V*_T_ at inflection point, mL	1058 (224)	+102 (167) *	1003 (212)	+118 (231)	0.9683
*f*_R_, breaths·min^−1^	30 (7)	+1 (5)	30 (6)	−3 (6) *	0.0415
T_i_/T_tot_	0.36 (0.05)	+0.01 (0.04)	0. 36 (0.07)	+0.02 (0.05)	0.6593
*V*_D_/*V*_T_	0.36 (0.08)	+0.00 (0.05)	0.36 (0.05)	+0.00 (0.05)	0.9415
HR, beats·min^−1^	122 (17)	+6 (12)	114 (21)	−1 (16)	0.4035
O_2_ pulse, mL·beats^−1^	5.6 (1.2)	+0.1 (0.7)	6.1 (1.6)	−0.5 (0.6) **	0.0108
SpO_2_, %	90.3 (4.7)	+0.1 (2.8)	89.3 (5.6)	−0.9 (3.1)	0.1723
PetCO_2_, mmHg	37.5 (6.3)	−1.0 (2.8)	37.4 (4.7)	−0.4 (4.1)	0.5200
ΔFO_2,_ %	2.85 (0.50)	−0.10 (0.34)	2.89 (0.41)	−0.03 (0.37)	0.4558
*V’*_E_/*V’*_CO2_	43.2 (8.3)	+1.8 (4.9)	42.8 (7.1)	+0.6 (4.2)	0.2792
*V’*_E_–*V’*_CO2_-intercept, L·min^−1^	6.7 (2.7)	−0.2 (2.0)	5.9 (3.3)	−1.2 (2.4) *	0.1883
*V*_D_-intercept/*V*_T_	0.44 (0.20)	−0.01 (0.37)	0.41(0.27)	0.03 (0.22)	0.6674
*V’*_E_/*V’*_CO2_-nadir	42.9 (8.3)	+1.2 (4.4)	42.4 (7.1)	+0.8 (4.1)	0.5685
*V’*_E_–*V’*_CO2_-slope	32.8 (8.5)	+2.6 (4.6) *	33.9 (10.2)	+2.7 (4.5) *	1.0000
Time slope, sec·mL^−1^·min	0.96 (0.28)	+0.07 (0.30)	0.98 (0.34)	+0.19 (0.25) **	0.3310
The causes to stop during CPET	Pre-PR	Post-PR	Pre-PR	Post-PR	Not done
Dyspnea/leg fatigue	14/6	11/9	11/7	9/9	Not done

Data are presented as means (standard deviation). Peak *V’*_E_: minute ventilation at peak exercise; inc.: increase; PR: pulmonary rehabilitation; six-MWD: six-minute walking distance; CPET: cardiopulmonary exercise testing; *V’*_O2_: oxygen uptake; R: gas exchange ratio; *V*_T_: tidal volume; *f*_R_: breathing frequency; T_i_/T_tot_: inspiratory duty cycle; *V*_D_/*V*_T_: physiological dead space/tidal volume ratio; HR: heart rate; O_2_ pulse: *V’*_O2_/HR; SpO_2_: percutaneous oxygen saturation; PetCO_2_: partial pressure of end-tidal carbon dioxide; ΔFO_2_: difference between inspiratory and expiratory O_2_ concentrations; *V’*_CO2_: carbon dioxide output; *V*_D_-intercept: *V*_D_ calculated as *V’*_E_-axis intercept/*f*_R_-axis intercept, obtained from *V’*_E_ vs. *V’*_CO2_ and *f*_R_ vs. *V’*_CO2_ relationships (see the Methods section for details); *V’*_E_/*V’*_CO2_ nadir: the lowest value of the ratio between *V’*_E_ and *V’*_CO2_ during exercise (see the Methods section for details); *V’*_E_–*V’*_CO2_ slope: the slope was determined by linear regression analysis of *V’*_E_ to *V’*_CO2_ obtained during exercise (see the Methods section for details); Time slope: ratio of exercise time until exhaustion to Δ*V’*_O2_ (peak minus resting *V’*_O2_) during CPET (see the Methods section for details). * *p* < 0.05, ** *p* < 0.01, *** *p* < 0.001: compared with pre-PR values (within-group difference). ^†^ The data of 6-MWD were not obtained from two patients. ^††^: 27 patients (peak *V’*_E_ inc. group, *n* = 15; peak *V’*_E_ non-increase group, *n* = 12), whose anaerobic thresholds were obtained before and after pulmonary rehabilitation were analyzed.

**Table 6:** Correlations between the change in the ΔFO_2_ at peak exercise resulting from pulmonary rehabilitation and other parameters (*n* = 38).

	r	*p*-Value
Dyspnea at peak exercise diff., Borg scale	0.1361	0.4153
Peak *V’*_O2_ diff., mL·min^−1^	0.4884	0.0019
*V’*_O2_ at anaerobic threshold diff., mL·min^−1^	0.6711	0.0001
*V’*_E_ at peak exercise diff., L·min^−1^	−0.0988	0.5552
*V*_T_ at peak exercise diff., mL	0.2655	0.1072
*f*_R_ at peak exercise diff., breaths·min^−1^	−0.3894	0.0157
*V*_D_*/V*_T_ at peak exercise diff.	−0.2428	0.1419
O_2_ pulse at peak exercise diff., mL·beats^−1^	0.2547	0.1228
*V’*_E_/*V’*_CO2_-nadir diff.	−0.7057	<0.0001
*V’*_E_–*V’*_CO2_-slope diff.	−0.4578	0.0039
Time slope diff., s·mL^−1^·min	−0.4518	0.0044

ΔFO_2_: difference between inspiratory and expiratory O_2_ concentrations; Diff.: value after pulmonary rehabilitation minus the value before pulmonary rehabilitation; *V’*_O2_: oxygen uptake; *V’*_E_: minute ventilation; *V*_T_: tidal volume; *f*_R_: breathing frequency; *V*_D_/*V*_T_: physiological dead space/tidal volume ratio; O_2_ pulse: *V’*_O2_/heart rate; *V’*_CO2_: carbon dioxide output; *V’*_E_/*V’*_CO2_-nadir: lowest value of the ratio between minute ventilation and carbon dioxide output during exercise (see the Methods section for details); *V’*_E_–*V’*_CO2_-slope: the slope was determined by linear regression analysis of minute ventilation to carbon dioxide output obtained during exercise (see the Methods section for details); Time slope: ratio of exercise time until exhaustion to obtained Δ*V’*_O2_ (peak minus resting *V’*_O2_) during cardiopulmonary exercise testing (see the Methods section for details).

## Data Availability

The data that support the findings of this study are available from the corresponding author upon reasonable request.

## Abbreviations

CPET: cardiopulmonary exercise testing; ΔFO_2_, difference between inspiratory and expiratory oxygen concentrations; GOLD, Global Initiative for Chronic Obstructive Lung Disease; SAMA, short-acting muscarinic antagonist; LAMA, long-acting muscarinic antagonist; SABA, short-acting β2-agonist; LABA, long-acting β2-agonist; ICS, inhaled corticosteroid.
